# Wound management and healing in space

**DOI:** 10.3389/fbioe.2022.958515

**Published:** 2022-08-29

**Authors:** Christopher Puhl, Nicol Caplin, Anna Fogtman, Angelique Van Ombergen

**Affiliations:** ^1^ Telespazio Belgium S.R.L. for the European Space Agency, Noordwijk, Netherlands; ^2^ SciSpacE Team, Directorate of Human and Robotic Exploration Programmes, European Space Agency (ESA), Noordwijk, Netherlands; ^3^ Space Applications Services NV/SA for the European Space Agency, European Astronaut Centre, Cologne, Germany

**Keywords:** wound healing, tissue engineering, wound management, 3D bioprinting, space exploration

Wound healing allows the body to repair damage by regenerating the skin barrier, protecting the skin from the external environment, and allowing it to maintain its homeostasis. It is a complex and multi-step process involving a set of biological mechanisms that should follow a specific sequence, modality, and timeline. Any alterations to this, endogenous or exogenous, can lead to impaired healing which could result in the formation of scar tissue or other pathological conditions (Wilkinson and Hardman, 2020).

The space environment comes with environmental conditions (such as microgravity and ionising radiation) and several constraints in terms of available medical care. Previous studies (Locatelli et al., 2021; Morbidelli et al., 2021; Riwaldt et al., 2021; Marvasi et al., 2022) have already shown that wound healing is slowed down and impaired in space due to the unloading related to the microgravity environment.

Additionally, the combined effects of different stressors of spaceflight, including isolation, a hostile environment or ionising radiation (IR) can further impact an increased susceptibility to infection and delayed wound healing (Chancellor et al., 2018). IR can be a single showstopper to deep space exploration, and it was previously demonstrated that repetitive irradiation impairs cellular replication, causes chronic inflammatory responses, and disturbs proliferation. This leads in consequence to many acute and long-term effects and delayed wound healing is one of them. Skin atrophy, soft tissue fibrosis and microvascular damage has been observed in patients undergoing radiotherapy, and those effects increase the likelihood of impaired tissue regeneration (Jacobson et al., 2017).

As humans venture deeper into space, with longer missions and limited medical capabilities, as well as the higher risk of acute radiation effects due to Solar Particle Events (SPE), investigating wound healing mechanisms adds to the list of knowledge gaps in space research.

Furthermore, a serious risk of long-term space travel and exploration is the potential for the space crew to sustain injuries. The inability to plan for every possible situation, and the lack of a hospital or specialised medical care, means that even a relatively small injury can become serious and potentially threaten the life of the crew and the long-term viability of a given mission. It is critical to address this concern to be prepared for longer-term and deep space missions.

The know-how and applications stemming from this research will have a significant impact on terrestrial medicine, as the problem of insufficient medical resources has been presented in the frame of an exponentially growing human population, more often populating remote areas with access difficulties. Lastly, relocation of large human populations due to climate change will call for novel medical applications for treatment in remote locations and understanding the mechanism of wound healing is a crucial step in this process.

There are several important considerations (non-exhaustive list):

1. Space exploration requires careful planning for payloads. Limited space and other constrained resources make it difficult (and at times impossible) to give medical care in accordance with terrestrial standards.
2. Transporting medical grade materials from Earth into space potentially requires temperature control and the fragility of these materials also needs to be considered. The extreme environmental factors in space will require developing new ways of storing medical supplies and pharmaceuticals to ensure their survival. Potential new materials with adjusted properties need to be developed to ensure they are robust enough for application in space.
3. The physical properties of matter are altered in microgravity, therefore standard ways of drug storage and administration are often not sufficient in spaceflight.
4. The number of possible medical problems that might arise during long-term space exploration is vast. Future mission scenarios involve crew travelling deeper into space and therefore further from help if required. As the temporal aspect of wound management can be critical, novel ways of conducting this in space will be a necessity.
5. The understanding of the mechanisms underlying wound healing in space, and how these are altered remains to be accurately identified.

Consequently, the topic of wound healing in the context of spaceflight has been approached by ESA in a similar fashion to other potentially mission critical problems. ESA has set up Topical Teams—think tanks of experts and leaders in a particular field with the goal of identifying potential concerns and developing recommendations to address these using practical solutions.

The culmination of the Topical Team “*Tissue Healing in Space: Techniques for Promoting and Monitoring Tissue Repair and Regeneration*” is the series of articles in this special issue, as well as several other ongoing and planned activities. In June 2022, ESA’s *Suture in Space* experiment is scheduled for flight on the International Space Station (ISS). This experiment aims to investigate the behavior of sutured wounds and the repair process in microgravity, using two models of sutured wound healing based on tissue cultures from human skin and blood vessels, respectively. The *Suture in Space* experiment will lead to improved knowledge of the molecular, cellular and tissue mechanisms involved in the healing of sutured wounds, as well as yield important information to develop strategies to manage wounds in space and promote healing, both in space and on Earth (Monici et al., 2021). ESA also has on-going activities in this area in its Microgravity Application Programme (MAP), i.e., WHISKIES and WHISPER. Both projects grew out of the Topical Team and focus on space environment induced changes to biomedical materials for wound treatment and the fundamental biological wound healing mechanisms.

ESA is also building a 3D bioprinting and 3D cell maturation capability in Low Earth Orbit which will provide support for research and preparation activities to enable long-term human deep space exploration. This will enhance our fundamental understanding and characterisation of the effects of space stressors. Looking further into the future, 3D bioprinting offers the potential to generate personalised grafts or implants for repair of tissue injuries for crew members during long-term deep space exploration missions, where a rapid return to Earth is not possible (Cubo-Mateo and Gelinsky, 2021).

There are still many unknowns, but we are convinced that the contributions to this special issue provides the current state of the art, as well as important next steps and fascinating foresights for wound healing in the context of space exploration. Without question will the knowledge gained from these activities not only advance space research and human exploration, but also deliver answers to fundamental questions in the area of wound healing and yield translational applications to terrestrial medicine related to trauma care and emergency surgery amongst other fields.

Lastly, we would like to thank the editors and the authors for their efforts in compiling this vital work, as well as the many dedicated peer reviewers for reinforcing the excellence in the quality of the papers. A special thanks goes out to Prof. dr. Monica Monici, whose dedication and contributions have been pivotal in these efforts and activities.
